# Action-oriented prospective policy analysis to inform the adoption of a fiscal policy to reduce diet-related disease in the Solomon Islands

**DOI:** 10.1093/heapol/czab031

**Published:** 2021-04-07

**Authors:** Erica Reeve, Anne Marie Thow, Salome Namohunu, Colin Bell, Anita Lal, Gary Sacks

**Affiliations:** Deakin University, Geelong, Global Obesity Centre, Institute for Health Transformation, School of Health and Social Development, 1 Gheringhap Street, Geelong, VIC 3220, Australia; Menzies Centre for Health Policy, School of Public Health, University of Sydney, Level 2, Charles Perkins Centre, University of Sydney, NSW 2006, Australia; Solomon Island Government, Ministry of Health and Medical Services, Honiara, Solomon Islands and UNICEF Pacific Islands; Deakin University, School of Medicine, Faculty of Health, Geelong, Global Obesity Centre, School of Health and Social Development, 1 Gheringhap Street, Geelong, VIC 3220, Australia; Deakin University, Geelong, Global Obesity Centre, Institute for Health Transformation, School of Health and Social Development, 1 Gheringhap Street, Geelong, VIC 3220, Australia; Deakin University, Geelong, Global Obesity Centre, Institute for Health Transformation, School of Health and Social Development, 1 Gheringhap Street, Geelong, VIC 3220, Australia

**Keywords:** Prospective policy analysis, implementation, fiscal policy, SSB tax, Pacific Islands, noncommunicable disease

## Abstract

Fiscal tools are recommended as a part of a comprehensive approach to diet-related disease prevention, however, widespread adoption has been hampered by political and economic resistance. The aim of this study was to support an advocacy coalition in the Solomon Islands with evidence-based consideration of the development and implementation of a tax on sugar-sweetened beverages (SSBs), sensitive to local contextual factors and constraints. In 2017–19, we conducted a prospective policy analysis, including document analysis and qualitative interviews with key stakeholders to elicit policy-relevant data, a quantitative analysis to frame the policy problem and examine appropriate implementation mechanisms, and economic modelling to outline the potential benefits associated with different proposed policy solutions. Applying an action-oriented approach to prospective policy analysis enabled us as researchers to engage in the needs of a ‘pro-SSB tax’ advocacy coalition and prepare them to exploit policy opportunities created by the meeting of policy ‘streams’. Our analysis demonstrated that SSBs were being consumed in relatively large amounts, especially by children, and that there were likely to be substantial health and economic benefits associated with a SSB tax. Increasing fiscal uncertainty for key sectors had created an environment prime for the advocacy coalition to pursue the adoption of an SSB tax. However, we found that policymakers face a number of practical challenges in securing effective adoption and implementation of global food policy recommendations, including that it is difficult to demonstrate the potential efficacy of interventions in the local context. The development of a policy package based on local factors resulted in a policy product that was likely to be more persuasive for local policymakers and policy leaders. We suggest that there is substantial scope for researchers to more effectively engage with policy advocates to inform and shape real-world health policy improvements.

**Key Messages**

- Securing implementation of global food policy recommendations requires local evidence, but it may be difficult in data-poor settings to demonstrate the potential efficacy of interventions in the local context.
- An action-oriented approach to prospective policy analysis can enable researchers to engage in the needs of public health advocacy coalitions and prepare them to exploit policy opportunities.
- Increasing fiscal uncertainty in lower-income countries may create an environment prime for public health advocacy coalitions to pursue the adoption of a sugar-sweetened beverage tax, which is likely to have clear economic and health benefits.

## Introduction

Noncommunicable diseases (NCDs) are the leading cause of mortality globally (World Health Organization, 2018). NCDs are more prevalent in lower-income countries, where they have major impact on economic development and livelihoods (Nugent, 2018). Global trends indicate that consumption of sugar-sweetened beverages (SSBs), a major source of free sugar intake globally, has increased significantly over recent years, driven by rapid globalization and urbanization (Popkin, 2006; Baker and Friel, 2016), and pervasive food marketing to children (World Health Organization, 2009). Increasing affordability of SSBs has been a major influence on these trends (Blecher *et al.*, 2017; Ferretti and Mariani, 2019), with affordability increasing more rapidly in lower-income settings (Blecher *et al.*, 2017). Dietary changes in the Pacific Islands, including rapid increases in consumption of processed foods and beverages, have contributed to increasing prevalence of NCDs (Snowdon, 2014; Hawley and McGarvey, 2015). NCDs are now the leading cause of mortality in Pacific Island Countries (Hoy *et al.*, 2014).

The World Health Organization (WHO) Global Action Plan for the Prevention and Control of Non-communicable Diseases (2013–20) calls on countries to consider fiscal tools (taxes and incentives) that encourage consumption of healthier foods and discourage less healthy options as a part of a comprehensive response to addressing diet-related NCDs. In addition, WHO recommends limiting free sugar intake to no >10% of total energy (World Health Organization, 2015). International evidence suggests that SSB taxes are an effective mechanism for reducing consumption of SSBs (Thow *et al.*, 2014), and can lead to significant improvements in population diet (Cabrera Escobar *et al.*, 2013). Modelling studies from several countries have suggested that a tax on SSBs is likely to be cost-effective and beneficial to government revenue (Long *et al.*, 2015; Manyema *et al.*, 2016; Veerman *et al.*, 2016).

In the Solomon Islands, 69% of all deaths are attributable to NCDs (World Health Organization, 2018). In response to this, the Ministry of Health and Medical Services (MHMS) and the Ministry of Finance and Treasury (MOFT) identified SSB taxes as a priority policy intervention for reducing the burden of NCDs in the Multi-sectoral NCD Strategic Plan (2019–23) (Solomon Islands Ministry of Health and Medical Services, 2017), and as an opportunity to generate additional revenue as a part of a major tax reform process (Solomon Islands Government, 2017). However, capacity to drive and implement preventative nutrition policies in the Solomon Islands is impaired by limited health systems capacities, and the sheer number of pressing health priorities (World Bank, 2018).

For countries like the Solomon Islands, the process of developing effective multisectoral policies has proved challenging (Gilson and Raphaely, 2008; Pelletier *et al.*, 2018). Even for employees of the state, policy and decision-making processes can be ‘opaque’, and sourcing the required evidence to navigate the process can be problematic (Walt *et al.*, 2008). In the Pacific Islands, the high cost of conducting national food surveys (Parry, 2010) and limited capacity for data analysis (Lum *et al.*, 2009) makes it difficult to develop compelling, evidence based policy proposals. In addition, the design and adoption of an SSB tax requires many decisions (Chriqui *et al.*, 2013), including the point at which the tax is collected (e.g. importer, manufacturer, retailer), the tax type (e.g. percentage increase in price, volume or sugar), the beverages to be subjected to, or excluded from, taxation, the tax rate, and what should be done with the tax revenue. Decisions made during policy design result in trade-offs with regards to cost, equity, efficiency, quality in a particular context (Thow *et al.*, 2018).

As an increasing number of countries consider the implementation of SSB taxes, there are emerging opportunities to conduct research that systematically supports countries through these policy problems (Pelletier *et al.*, 2013). Policy analysis is a well-established approach explaining the interactions between interests, ideas and institutions as they relate to a policy, with strong utility for exploring contextual factors in which policies are implemented (Walt *et al.*, 2008). Much health-related policy analysis has been retrospective and focused on successes or failures in past policies. In contrast, there are few accounts of forward-looking policy analysis involving real-time documentation and lesson-drawing to inform policy processes (Pearson *et al.*, 2010). Applied prospectively, policy analysis can be utilized to formulate an effective response to policy problems before policy actions are carried out by developing a nuanced view of the policy problem, and providing timely advice towards meeting policy objectives that are sensitive to local contextual factors and constraints (Buse, 2008). In addition, prospective policy analysis can be used to reveal policy windows and to identify the information and resources required to drive policy change in a particular setting (Buse, 2008). Prospective policy analysis research can take the form of ‘action-oriented research’ when it includes an emphasis on engagement and knowledge translation to support policy change (Buse, 2008). While the potential value of action-oriented research in the area of nutrition has been recognized, it is underrepresented in the nutrition literature (Pham and Pelletier, 2015).

The aim of this study was to conduct action-oriented prospective policy analysis to support evidence-based consideration of the development and implementation of a tax on SSBs in the Solomon Islands.

## Materials and methods

### Overarching study design

In 2017–19, we applied an action-oriented approach for supporting advocacy for a tax on SSBs in the Solomon Islands. The study involved a mixed-method prospective policy analysis which included a document analysis, a quantitative analysis to identify potential impacts of SSB taxes on population health, and qualitative interviews to elicit policy-relevant data (Walt *et al.*, 2008; Gilson, 2014). We used this information to develop an evidence-based policy proposal for an advocacy coalition, sensitive to local contextual factors and constraints. These methods are described in more detail in the sections that follow.

### Policy engagement activities

The research involved extensive engagement with members of an ‘advocacy coalition’ (Sabatier, 2007), led by collaborators from the MHMS in the Solomon Islands, development partners from the Food and Agriculture Organization of the United Nations (FAO), WHO, and the Secretariat of Pacific Communities (SPC)(a Pacific regional technical organization). Our engagement was initiated in 2017 when the implementation of a SSB tax was identified by MHMS as a priority, and FAO engaged us to develop evidence supporting an SSB tax and to inform tax design. We began our engagement via email and telephone with FAO and MHMS, culminating in our first data collection visit in February 2018 (2 weeks). We visited again in July 2019 (2 weeks) and September 2019 (3 days) to support the advocacy coalition with work on their broader food and nutrition security agenda.

In 2017, policy engagement activities suggested that generating an economic health model to estimate the potential fiscal and health-related benefits of implementing a SSB tax in the Solomon Islands would strengthen the work of the advocacy coalition. At this time, the advocacy coalition indicated that they were requiring a document outlining options for defining and targeting SSBs for taxation (including powder mixes, e.g. sweetened powder mixes designed to be diluted with water to produce beverages), with an articulation of the implications of different implementation mechanisms. The information requirements of the advocacy coalition, as determined through our policy engagement activities, shaped the tasks carried out as part of the research.

### Theoretical framework

We drew on a well-established theory of the policy process and policy change for all aspects of the study (Walt and Gilson, 1994; Walt *et al.*, 2008; World Health Organization, 2012). In particular, we applied key constructs from Kingdon’s Multiple Streams Framework (MSF) (Kingdon, 1984) because of its relevance to the study aim. The MSF suggests that simultaneously introducing a policy problem and policy solution to the attention of policy leaders at a time when political appetite is conducive may enhance likelihood of policy change (Kingdon, 1984). Policy windows are thought to emerge when these streams align to form political windows, but they do not guarantee the passage of a policy (Howlett *et al.*, 2013). We also drew on the Advocacy Coalition Framework (ACF) when an informal network of interested parties emerged. Application of the ACF prompted us to think about the needs, interests and capacities of the policy community to advance this agenda, and to identify other relevant coalitions, including those supporting or opposing the tax (Sabatier, 2007). ACF suggests that advocacy coalitions that are prepared to capitalize on opportunities for policy change when they arise are more likely to be successful (Buse, 2008; Shiffman and Smith, 2007). We aligned our work against the key constructs of the MSF to sensitize ourselves to the conditions in which policy change was likely, so that we could prepare the advocacy coalition with a policy proposal in the event that a ‘window of opportunity’ was presented (Buse *et al.*, 2005). These theories were used to make generalizations related to policy implementation that could be applied in other contexts (Walt *et al.*, 2008).

In our policy analysis, we conceptualized policy processes as part of a policy cycle heuristic (Howlett *et al.* 2009). Though policymaking is not always linear (Walt *et al.*, 2008), the policy cycle heuristic provided a broad sequence of events involved in policy processes, including the administrative steps to bring policy proposals to the attention of policy leaders, develop policy specifications and implement them (Cairney, 2013).

### Qualitative data collection

#### Documents

We reviewed 14 publicly available documents sourced through an internet search (using Google search engine) or provided by the advocacy coalition. Document analysis sought to identify framing of problems related to nutrition consumption and diet-related NCDs (the ‘problem’ stream), the emergence of food and beverage (and other health) taxes as a policy solution (the ‘policy’ stream), and to gain insight into the political context that might influence the likelihood of a policy being adopted (the ‘politics’ stream). Documents included health surveys (3), multisectoral food and health policies (3), sector-specific strategies (5), and documents contributing an understanding of the relevant trade and fiscal context, including fiscal legislation (2), trade agreements (2), government discussion papers and analyses (3) and the National Development Strategy (1).

#### Interviews with policy officers and stakeholders

In February 2018, we conducted semi-structured in-depth interviews with 18 stakeholders in the Solomon Islands, including members of the advocacy coalition. Interview schedules were developed based on the concepts of the selected theoretical framework. The interviews focused on identifying appropriate ways to frame the policy problem and policy solution as well as aspects of the political context that might influence the likelihood of a policy being adopted or opposed. In addition, the interviews sought to gather evidence surrounding food taxation implementation in the local policy context, for instance, agency roles and capacities, and influences on policy administration (e.g. coherence with food regulations).

Aligned with participatory research principles, interviews were arranged and attended by MHMS collaborators. Interviewees initially included all those identified as relevant to the policy process, with snowball sampling used to identify additional informants. We interviewed senior and mid-level policymakers from the MHMS (*n* = 5), the MOFT (*n* = 3), the Ministry of Commerce, Industry and Labour (*n* = 3), the Office of the Attorney General (*n* = 1), development partners engaged with MHMS and MOFT (*n* = 5), and a representative of a major manufacturer of SSBs in the Solomon Islands (*n* = 1). There were no NCD-related civil society advocacy groups active in the Solomon Islands at that time, and we could not reach representatives from the Chamber of Commerce.

Interviews were conducted in English and were between 10 and 90 min in duration. All interview participants provided informed consent to participate. We also sent every participant by email either interview notes (submitted within 24 h of their interview) or typed transcripts (where consent was given to record) for verification.

As is appropriate in action-oriented research, the interviews involved a two-way exchange. Participants themselves had queries about the policy options and process, and we documented these to ensure they were responded to throughout the research process.

### Coding and analysis

Data were coded deductively by one author, based on constructs from key theories of policy process including MSF, ACF and the policy cycle heuristic, using Nvivo software. These themes included: the process and requirements of policymaking in the Solomon Islands (policy cycle); evidence and framing of the policy problem (problem stream); evidence to inform or support the policy solution (policy stream); political for conditions for receptivity or rejection of policy change (politics stream); and evidence of a policy opportunity (policy windows). We added themes to gather information on: the needs and resources of the advocacy coalition to advance the policy process; influences on tax implementation mechanisms and structure; and evidence of an opposing advocacy coalition. A draft description of these themes was written and reviewed by two other authors. We triangulated qualitative information by comparing the interviews with the documentary data, and by cross checking results with the advocacy coalition.

### Quantitative analyses

Prior to our first visit to the Solomon Islands in 2018, we undertook quantitative analyses to generate evidence related to the policy problem and the potential solution. These included: (i) the calculation of baseline SSB consumption by Solomon Islanders; (ii) modelling of the likely effect of a SSB tax on population body weight, and the economic and obesity-related disease benefits of a SSB tax; and (iii) an estimation of the costs of implementing a SSB tax. Based on discussions with health policymakers and international literature, we modelled the impact of a 40% and 20% tax, and a 50% pass-through of a 20% tax (effectively a 10% price rise) (Nakhimovsky *et al.*, 2016; World Health Organization, 2016). Supplementary File S1 explains the methods and modelling in more detail.

#### SSB consumption and changes in weight

In the absence of national nutrition survey data, baseline SSB consumption was based on the NCD STEPS survey 2015 (Ministry of Health & WHO, 2017), the Global School Based Health Survey (GSHS) 2011 (World Health Organization, 2011) and the 2012/13 Household Income and Expenditure Survey (HIES)(Solomon Islands Government, 2015). For the proposal to demonstrate the contribution of sugar to dietary intake, we converted expenditure and acquisition data from HIES into a proxy of household food energy and nutrient intake to establish the nutrient and food energy values for each household member, by household type (Molteldo *et al.*, 2014; Reeve *et al.*, 2019). Quantities of each food item were converted to nutrient values using Pacific conversion tables (Dignan *et al.*, 2004) and nutrient composition values identified through a 2011 survey of packaged foods in Samoa (Snowdon *et al.*, 2013).

**Figure 1:**
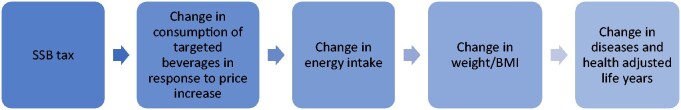
Logic model for health effects of SSB tax

We drew from international literature to demonstrate the way in which NCD risk-factors are mediated by dietary patterns (Alberti *et al.*, 2005), and to outline the potential contribution of SSBs to NCD risk (World Health Organization, 2015). In the absence of Pacific-relevant price elasticities, factor of −0.9 was used to estimate changes in consumption, informed by the international literature (Cabrera Escobar *et al.*, 2013; Nakhimovsky *et al.*, 2016) and guidance from The Pacific Community (Teng, 2015). The reductions in quantities of SSBs consumed were converted to kilojoule equivalents using nutrient tables for Australia (NUTTAB, 2010). Estimated changes in body weight for adults were calculated based on published relationships between changes in energy expenditure and body weight at the population level (Hall *et al.*, 2011). Figure 1 illustrates the logic pathway for measuring the expected impact of the tax from an obesity perspective.

#### Modelling of health and economic benefits

Obesity-related disease impacts associated with the proposed tax were estimated by adapting a model developed for Australia to determine the cost-effectiveness of obesity prevention interventions, including a SSB tax (Lal *et al.*, 2017). The model estimated the differences in life expectancy and health-adjusted life years pre- and post-implementation of the tax, based on differences in predicted variations in nine diseases caused by obesity. The model incorporated local population demographics and disease mortality and morbidity (SPC Statistical Division, 2013; Institute for Health Metrics and Evaluation, 2016), with case-fatality rates adjusted by a factor of 2.6 due to the difference in the probability of dying between ages of 30 and 70 in the Solomon Islands compared to Australia.

To calculate tax revenue, consumption volumes were drawn from household acquisition data from the HIES 2013 (Solomon Islands Government, 2015). Product prices were obtained by collaborators from MHMS who undertook an audit of store pricing of some of the most commonly available SSBs in Honiara.

Due to data limitations, comprehensive estimation of the healthcare costs savings was not possible. However, we were able to calculate the potential savings from hospital admissions of cases of diabetes avoided based on estimates of an average cost per admission in the Solomon Islands (Lorgelly *et al.*, 2015). We also estimated the administrative costs associated with implementation of a new tax based on U.S. estimates (Long *et al.*, 2015) and local salaries (Strategic Pay Ltd, 2016).

We present, below, our findings organized in terms of the three streams of the MSF, and provide a summary of how the provision of this policy advice supported the work of the advocacy coalition.

## Result

### Politics stream: SSB taxes were palatable in the current political and economic climate

Our document analysis found that the Solomon Islands faced poor economic growth prospects in the context of a post-conflict environment with a declining resource base (World Bank, 2018). Economic and social development was the basis of the Medium-Term National Development Strategy (2016–20). A rapidly growing population, and scaled-down development partner assistance to the health sector, had reduced available budget for health (World Bank Group, 2019). In response to these challenges, in 2017 political leaders had spurred a major economic reform process seeking opportunities for more efficient and equitable revenue collection (Solomon Islands Government, 2017), which emphasized consumption taxes and the identification of more reliable revenue opportunities.

During our interviews in 2018, MHMS policy leaders expressed that they believed that the current Prime Minister and Minister of Finance would be relatively supportive of SSB taxes should they be provided information on the associated health and economic benefits. Additionally, we found broad support for the tax among the four government agencies collectively responsible for economic reform namely, inland revenue, customs and excise, and commerce and industry. Representatives were consistent in their concerns about sugar consumption and NCD rates in the Solomon Islands, and worried about ‘excessive’ consumption of SSBs by the population.

*Rates of NCDs in Solomon Islands are alarming and there is support from MCIL for taxes aiming to reduce rates of NCDs and save health care expenditure* (Interview 19, Official from Ministry of Commerce, Industry and Labour).

Our interviews also revealed significant regional cooperation on the issue of SSB taxes, with Pacific island countries being supported on this issue by WHO, FAO, The Pacific Community and the World Bank.

*We've got a big focus on reducing things like obesity and cardiovascular disease. It's really high on the agenda* (Interview 11, Development Partner).

An opposing coalition was not identified during our research. Participants explained that the lack of opposition from the private sector was possibly because both local SSB producers also manufacture alcohol products, and therefore SSBs are less integral to their business outcomes. That said, the SSB producer we interviewed was of the view that an SSB tax would unfairly target their products, and that behavioural approaches targeting bread, rice and exercise would be more effective in reducing NCDs.

### Problem stream: SSB consumption was recognized as a contributor to high rates of diet-related NCDs

There was substantial attention given to the problem of poor diet among government policy documents. The National Health Strategic Plan (2016–20) noted a need to prioritize nutrition given high rates of undernutrition in under 5’s and escalating prevalence of diet-related NCD risk factors. The country’s National Food Safety, Food Security and Nutrition Plan (2019) had linked consumption of sugary foods to NCDs.

Apart from the SSB manufacturer we interviewed, all interviewees identified SSB consumption as a concern to them, particularly by children and adolescents. Policymakers reported increases in the variety and volume of SSB products available in the country, including in small remote stores and schools, and the persistent marketing of them via billboards, signs and in-store promotions.

*I think it's [SSB consumption] increasing day by day because of the number and the quantities of soft drinks coming into the Solomon Islands. Every month you can see new drinks coming into Solomon Islands… And you can see in the schools as well that there are lots of sugary drinks that are sold* (Interview 27, Nutrition Official).

Our quantitative analysis indicated that SSB consumption was highly prevalent, and could thus be considered an NCD risk factor in the Solomon Islands. Our estimates of energy and nutrient availability from the analysis of the HIES survey found SSBs and frozen ices (‘ice-stick’ or ‘ice-cup’) were contributing to 4.3% and 1.8% of total energy intake respectively, and that the average Solomon Islander consumed around 20% of their total daily energy intake from foods that were ‘high in sugar’, well in excess of recommendations. Our analysis also demonstrated that SSBs did not contribute significantly to intakes of protein or vitamin A or vitamin C, though we found that Milo™ contributed around 10% of total iron intake due to its fortification. Indeed, interviewees noted that it was important to ensure that interventions recommended to reduce consumption of sugar did not inadvertently exacerbate nutrient deficiencies, particularly for vulnerable groups.

The HIES analysis showed that powder mixes such as ‘3-in-1 coffee and tea mixes’ (pre-prepared mixes of instant coffee/tea, powdered milk and sugar) were being consumed by Solomon Islanders in large quantities, at around six times the volume of carbonated beverages. Carbonated beverages, frozen ices and sugary drink mixes (e.g. Tang) were also being consumed in relatively large amounts (Table 1). The STEPS survey reported adults were on average consuming two serves of SSBs per week (Solomon Islands Ministry of Health and Medical Services & World Health Organization, 2017). Younger adults (aged 18–29) were consuming an average of 2.6 serves of sugary drinks per week, double the amount of those in older age brackets (45–69) (1.3 serves per week).

**Table 1:** Estimated revenue generation from a SSB tax in the Solomon Islands at different tax rates

	Annual Household acquisition (g)	Total consumption after 20% tax^a^	Tax revenue at 20% tax (SBD)	Total consumption after 40% tax^a^	Tax revenue at 40% tax (SBD)
			$4/l (liquid SSB)^b^ $0.03/g (powder SSB)^b^ $0.01/g (ices)^b^		$8/l (liquid) $0.06/g (powder) $0.02/g (ices)
3-in-1 powder (coffee, tea)	343 957 014	282 044 751	7 897 253	220 132 488	12 327 419
Carbonated soft drinks	529 536 671	434 220 070	1 823 724	338 903 469	3 647 448
Frozen ices	335 488 801	275 100 816	3 301 209	214 712 832	6 602 419
Juice drinks, cordial, flavoured powders	112 627 045	92 354 176	387 887	72 081 308	775 775
Chocolate powder drinks	40 386 522	33 116 948	927 274	25 847 374	1 854 549
Total (SBD)^c^	1 361 996 053	1 116 836 763	14 337 349	871 677 473	25 207 611
Total (USD)	163 439 526	134 020 412	1 720 482	104 601 297	3 024 913

a Based on price elasticity of demand for SSBs of −0.9.

b Modelling rate established based on surveyed price for that type of SSB.

c SBD = 0.12 USD (as at February 2018).

### Policy stream: SSB taxes are framed as beneficial and feasible

We found that all three of the country’s national frameworks for action on NCDs, food and nutrition [including the Kaikaim Lokol Kaikai (2019–23), the Multi-sectoral National Noncommunicable Disease Strategic Plan (2019–23) (Solomon Islands Ministry of Health and Medical Services, 2017) and the (draft) National Food Security, Food Safety and Nutrition Policy (2019–23)] identified SSB taxation as a promising policy to incentivise healthier consumption. The MHMS undertook scoping work to examine the possibility of adopting an SSB tax in 2016, following a meeting between policy leads at the MHMS and MOFT. However, according to interviewees, policymakers were not ‘ready’ to proceed with adopting the tax at that time due to a lack of capacity to address some of the technical issues associated with establishing a new tax, for example, the need to generate evidence on the likely effectiveness of a tax.

*Because last time [in 2016] when we went to do that, when we went for the meeting with Ministry of Finance on the tax thing on SSB, they were also waiting for evidence* (Interview 17, Development Partner).

*[The problem for us in designing a tax is] capacity I think…because this will be kind of technical. So maybe we need people to be really looking at this. If we leave it to anybody, no one can pick it up* (Interview 10, Health Official).

Our modelling exercise identified substantial potential health benefits associated with a SSB tax, largely attributable to reductions in incident cases of type 2 diabetes (Table 2). A 40% tax was likely to avert >24 000 cases of type 2 diabetes and substantially reduce cases of osteoarthritis, heart disease, stroke and cancer. Though a 20% tax rate would still have substantial health impacts, a reduced ‘pass-through’ rate (the extent to which the tax was passed on to consumers through increased prices) would compromise its impact suggesting that the tax level should be at least 20% to ensure benefits that they considered to be substantial.

**Table 2:** Mean SSB consumption in the Solomon Islands (2012), modelled change in SSB consumption, change in weight and impact on health outcomes

Modelled tax rate	Mean intake SSBs per person (g/day)	Change in SSB consumption (g/day)	Change in weight (kg)	Total HALYs gained (95% UI)	Lifetime change in incident cases for existing population^a^			
					Cancers^b^ (95% UI)	Heart disease and stroke^c^ (95% UI)	Type 2 Diabetes (95% UI)	Osteoarthritis (95% UI)
10% (50% pass-through)	88	−4.1	−0.06	312 (265 to 367)	−35 (−99 to 12)	−322 (−364 to −280)	−6517 (−7632 to −5392)	−3833 (−4888 to −2707)
20% tax	88	−8.0	−0.13	606 (510 to 724)	−69 (−194 to 20)	−623 (−701 to −550)	−12 692 (−14 822 to −10 519)	−2009 (−2567 to −1438)
40% tax	88	−15.2	−0.24	1149 (973 to 1360)	−132 (−363 to 35)	−1180 (−1320 to −1056)	−24 138 (−28 080 to −20 083)	−1027 (−1327 to −722)

Negative numbers indicate a reduction. HALYs, health-adjusted life years; UI, uncertainty interval; SSBs: Sugar sweetened beverages.

a Based on 2013 population size.

b Cancers include colon, breast, endometrial and kidney.

c Heart disease includes hypertensive heart disease, ischaemic heart disease.

Our modelling indicated that the potential revenue raised by an SSB tax would be substantial for the government at both a 20% and 40% tax rate (Table 1), with Solomon Islands Dollar (SBD) 14 337 349 or SBD 25 207 611 being raised annually (respectively). For comparison, the amount of revenue predicted to be raised through a 20% tax rate would be equal to the annual budget of the Ministry of Fisheries and Marine Resources in 2019 (Solomon Islands Government, 2019a).

With regard to policy administration, in our first visit (February 2018) we found that the Solomon Islands Government were reviewing options to improve efficiency and administration of their complex tax system, which included a goods tax, sales tax, import tax and a manufacturer excise. We found that foods and non-alcoholic beverages were already subject to import, excise and goods taxes. Imported foods were being taxed on entry under a new global customs management system, which automates foreign trade procedures. We also found that an excise tax system was already in place for alcoholic beverages and tobacco. With both of the local SSB manufactures already paying excise tax on the manufacture of alcohol products, an additional tax was deemed likely to be administratively straightforward to introduce. We found that a tax exemption was being applied to products originating in Melanesian countries as per the Pacific Island Countries Trade Agreement, and an agreement under the Melanesian Spearhead Group. This was deemed problematic due to the proportion of Coca Cola imported to the Solomon Islands from Papua New Guinea (another Melanesian country).

Interview participants indicated that it was necessary for the government to adopt a clear and non-discriminatory system of identifying which beverages are subject to tax and the point at which tax collection is to occur. Importantly, the system for identification of beverages for taxation needed to be transparent and non-discriminatory to meet the commitments of the Solomon Islands related to technical measures under the World Trade Organization. We found that the Food Control Regulations under the Pure Food Act (1996) mandated ingredient labelling of all food and beverage products (including sugar and other sweeteners), but they did not require disclosure of sugar contents (such as, sugar content per 100 g), meaning that a tax based on sugar thresholds would be difficult to implement effectively. In the absence of mandatory sugar content labelling on nutrient contents of packaged food, we found it would be possible to target SSBs through the Harmonized Commodity Description and Coding system (HS codes) used as a part of trade administration systems. The use of HS codes would make them identifiable through import manifest or excise declaration, without requiring packages to be opened to reveal food labels, or immediate changes to food labelling legislation. Based on this information, we identified five potential options for defining and targeting SSBs for taxation, including the potential implementation implications of each (Table 3).

**Table 3:** Design implications for SSB tax implementation in the Solomon Islands context

Policy options		Implications for implementation
1	Target *all products coded under a selection of HS codes*	Relatively simple to administer because of the adoption of the Automated System of Customs Data and thus the ability to ‘flag’ products for special treatment (e.g. special taxes). Requirements: Review of the HS codes to ensure that all ‘problematic’ SSBs are captured by targeted codes (e.g. beverages section and dairy section) ‘Splicing’ (disaggregation) of HS codes to avoid flagging non-SSBs within existing HS codes (e.g. sweetened coffee mix is targeted, but instant coffee is not), generating codes for all beverages with added sugar.
2	Target all beverages under a selection of HS codes *containing more than a certain percentage of sugar, as defined by a nutrient threshold*	Uses nutrient composition information to identify products containing sugar content above a designated threshold Requirements: Splicing of HS codes to define codes with sugar content above nominal threshold), with compliance checks by trained nutrition professionals Mandatory display of nutrient composition data to the exterior of shipping crates and cartons^a^ Training to enable customs officials to interpret nutrition composition panels and nutrient thresholds.
3	Target of all beverages with any ‘*added sugar*’ *or* ‘*free sugar*’ in the ingredients list	Uses ingredients lists to classify food. Requirements: Mandatory display of ingredients lists on manifest shipping crates and carton exterior^a^ Review of the HS codes to ensure that all ‘problematic’ SSBs were captured under a set of codes being targeted Training to enable customs officers to identify sugar-containing beverage products (i.e. sugar added under different ingredient names)
4	Apply a *volumetric tax based on sugar content by volume*	A variable tax based on sugar content and product volume Requirements: Mandatory display of nutrient composition data to the exterior of shipping crates and cartons^a^ Training to enable customs officers to undertake detailed calculations to determine the rate of tax
5	Apply an *ad valorem tax based on product value*	A tax applied to the product value outlined on the manifest Requirements: Splicing of HS codes to avoid flagging non-SSBs within existing HS codes (e.g. sweetened coffee mix is targeted, but instant coffee is not)

SSBs include all liquid and powdered beverages (including carbonated, milk-based, flavoured powders, cordials and juice drinks) that have been sweetened with any form of added sugar.

a These could possibly be addressed by requiring the exporting country to outline nutrition composition or ingredients lists of each product in the shipping manifest.

### Outcomes associated with policy engagement

In collaboration with partners from the Ministry of Health, we laid out a set of recommendations related to tax design (summarized in Table 4). Considering the administrative processes related to each tax option, and the existing tax structures imposed on tobacco and alcohol, the research team recommended that import and excise tax mechanisms might enable the most straightforward implementation, which was supported by the literature as an efficient method for food and beverage tax collection (Chriqui *et al.*, 2013). We recommended a ‘splicing’ (disaggregation) of HS codes to generate a series of subcodes specifically for beverages that are problematic from a public health perspective, as part of efforts to make identification of different SSBs easy for customs officials. We also recommended a range of complementary policy measures that would need to be implemented in order to increase policy effectiveness, and offered to support these activities. These included that trade-related tax exemptions be lifted from SSBs, that SSB taxes would need to be heavily promoted to members of the public, that the country adopt measures to monitor and communicate policy effectiveness to policy leaders, and that surplus tax excised be directed towards funding the provision of clean drinking water in schools.

**Table 4:** Summary of recommendations on SSB tax design

Objective of the tax	To reduce consumption of SSBs in the Solomon Islands, particularly in the population groups with the highest consumption (e.g. children and adolescents, urban dwellers), and prevent growth in consumption among the rural population
Definition and identification of beverages to be targeted	New HS codes are spliced (disaggregated) by a group of health and customs experts, so that all beverages with ‘added sugars’ are included. Added sugars can be identified through the ingredients list and declaration on manifest
Tax collection point	Excise is equally applied to imported and locally produced drinks by import excise and manufacturers excise, aligning to other ‘health taxes’—tobacco and alcohol excise
Rate of tax	20–40% Consider applying 20% to liquid beverages and 40% on powder beverages
Tax mechanism	A volumetric tax, applied as a tax per Litre (for liquids) or per gram (for powders) 20% tax is equal to: SBD 4/l or SBD 0.03/g 40% tax is equal to: SBD 8/l or SBD 0.06/g
Monitoring	Establish a SSB tax monitoring and evaluation plan to collect baseline and ongoing beverage pricing and consumption information, to convey trends in pricing, purchasing and tax revenue
Additional policy changes	Removal of import exemptions on SSBs to MSG countries, and the addition of SSBs to the exemptions list for PICTA Implementation of mandatory ingredients labelling, to be visible to customs officers on import

SSBs include all liquid and powdered beverages (carbonated, milk-based, flavoured powders, cordials and juice drinks) that have been sweetened with any form of added sugar. MSG: Melanesian Spearhead Group; PICTA: Pacific Island Countries Trade Agreement.

As part of our engagement activities we delivered an in-depth policy proposal, informed through the expressed needs of the advocacy coalition. This included: (i) the analysis of SSB consumption linked to NCD risk in the Solomon Islands; (ii) potential options for an evidence-based tax implementation mechanism and structure based on local and global evidence, with implementation implications; (iii) modelling of policy impacts; and (iv) mapping of policy process, including key steps to take.

We presented these results at a meeting with the advocacy coalition, together with our findings related to emerging political opportunities and policy barriers. Members of the advocacy coalition indicated that they recognized the opportunity, and that the data provided a compelling case based on local consumption and disease data.

*But having someone like you who collected this first will be a push to move forward……. So that's why this evidence that you're collecting now will be a very important step* (Interview 13, Health Official).

In 2018, the proposal was used by members of the advocacy coalition, including leaders at MHMS, WHO and FAO, to inform and develop proposed legislation, and to advocate the passage of the proposed SSB tax through Cabinet. In December 2018 the Public Accounts Committee formally recommended that the ‘Social Tax on Sugar’ be adopted by Parliament to address ‘alarming rates of diet-related diseases throughout the country’ (National Parliament of the Solomon Islands, 2018, p. 6). The Solomon Islands Government’s 2019 Budget Strategy Paper (Volume 1) confirmed that an SSB tax would be adopted as a measure to improve public health and increase revenue collection (Solomon Islands Government, 2019b). In 2020, members of the advocacy coalition reflected that the policy proposal we provided was the most helpful and meaningful work done to progress the adoption of a SSB tax. However, events of 2020 (including the pandemic) delayed implementation of the tax due to concerns around food security.

## Discussion

This study found that applying an action-oriented approach to prospective policy analysis enabled us as researchers to engage in the needs of a ‘pro-SSB tax’ advocacy coalition and support them with the development of an evidence-based policy proposal. The provision of timely advice helped to prepare them to exploit policy opportunities created by the meeting of policy ‘streams’ (Cairney and Zahariadis, 2016). We found that increasing fiscal uncertainty for key sectors created an environment prime for the advocacy coalition to pursue the adoption of an SSB tax in the Solomon Islands. The development of a policy package based on local factors resulted in a policy product with more persuasion for local policy makers and policy leaders, and assisted the advocacy coalition to capitalize on a policy window as it emerged. Based on the findings of this study and our experience gained from the action-oriented research process, we identified a number of policy lessons that are discussed, in turn, below.

### Nutrition policy development and implementation requires substantial analytic capacity

This study provided insight into practical challenges faced by lower-resource countries in advocating and implementing food policy commitments in alignment with global and regional health and development objectives. In this case, a lack of technical skills was identified as a key challenge facing policymakers, including difficulties demonstrating the potential efficacy of interventions in a compelling way (Thow, 2010), and operationalizing technical aspects tax design (Chriqui *et al.*, 2013; Thow *et al.*, 2018). After analysing the policy context, we found that different options had vastly different policy implications and outcomes requiring consideration. For instance, the literature suggests that volumetric tiered tax systems are most effective (Brownell *et al.*, 2009), but in the case of the Solomon Islands, this would not have been feasible due to the absence of sugar composition labelling, and would have been difficult to operationalize considering a large proportion of SSBs are purchased in powder form of varying dilutions. These findings are consistent with previous literature that has suggested that difficulties in compiling evidence to underpin policy advocacy efforts can be a barrier to policymaking with respect to public health (Hawkes *et al.*, 2016; Malla *et al.*, 2018), including in the Pacific Islands (Waqa *et al.*, 2017).

### Researchers can play an important role in actively supporting advocacy coalitions with ‘real-world’ food policy reform

This research demonstrated that action-oriented research provides an opportunity for researchers to engage more meaningfully to support policy advocacy in LMIC countries, by making explicit opportunities or options that may not be immediately apparent to policymakers (Hanney *et al.*, 2003). We demonstrated the value of applying policy theory to policy process by using MSF to sensitize the coalition to the emergence of opportunities (Kingdon, 1984), and then arming them with the requisite information to increase their chance of success (Shiffman and Smith, 2007; Buse, 2008). When we began working in the Solomon Islands, actors were not well organized. It was through application of ACF that the characteristics of a coalition were identified, and we saw opportunity to take advantage of external support for SSB taxes, and the apparent lack of opposition to them (Sabatier and Weible, 2014). This differed from the experiences of other countries, where health advocates have faced significant opposition to SSB tax from industry groups and government agencies with revenues that are linked to consumption (Thow *et al.*, 2010; Waqa *et al.*, 2017; Onagan *et al.*, 2019).

In addition, our modelling exercise was able to demonstrate that there was likely to be significant economic and health benefits associated with the adoption of an SSB tax in the Solomon Islands. The information our research provided addressed a key gap that had been identified by policymakers as a barrier to progress. For the Solomon Islands Government, SSB taxes could deliver a price that better reflects the health care costs associated with regular consumption (Thow *et al.*, 2018). These findings are consistent with reports that have argued that policies with demonstratable benefit across multiple government agencies and development priorities (economic stability and rising rates of NCDs), are more likely to gain traction (Thow *et al.*, 2011; Development Initiatives, 2017), though Pacific Islands Finance agencies had already demonstrated considerable commitment to addressing NCDs (2014) (Pacific Islands Forum Secretariat & Secretariate of Pacific Communities, 2014).

This study elucidated a number of other benefits associated with ‘research-to-practice’ partnerships (Buse, 2008). In particular, the action-oriented approach helped us to identify and respond to capacity challenges related to evidence generation and synthesis (Hawkins and Parkhurst, 2016). Undertaking the analysis prospectively meant that the research team could concurrently support the policy making process by engaging with the coalition and responding to their needs in a timely way. We made a substantial effort to be responsive to the queries, interests and concerns of the coalition, which is thought to increase prospects of overall success (Buse, 2008; Walt *et al.*, 2008). Drawing from the collective expertise of a multidisciplinary academic group (e.g. policy analysis, economics, dietary data analysis) enabled us to more rigorously and coherently explore and convey the policy problem and solution.

We sought to develop national capacity to undertake policy analysis and evaluation as a long-term strategy for accelerating the effective implementation of NCD prevention actions (Buse, 2008; Walt *et al.*, 2008). The involvement of the end-users (the advocacy coalition) in knowledge design and dissemination also contributed to a partnership of trust, ownership and consensus (Bell, 2009), one that is ongoing at the time of publication. This echoes findings from the literature that suggest that research co-design (including researchers and policymakers as part of the research process) can maximize the benefits gained through an ‘insider-outsider’ arrangement, by gaining the richest and most comprehensive understanding of policy processes (Walt *et al.*, 2008). Our findings also reinforce lessons learnt from previous studies that ongoing engagement between researchers and collaborators can help to overcome the commonly-faced tension between the short-term nature of research and funding, and the long-term nature of policymaking processes (Buse, 2008; Walt *et al.*, 2008). Our having spent extensive time in the policy subsystem will have increased our responsiveness to policy opportunities that often unfold slowly and over a long period (Sabatier, 1988), and reduced the burden normally associated with researchers being ‘orientated’ to a policy situation (Gilson *et al.*, 2018; Walt *et al.*, 2008). While efforts to implement the tax appear to have stalled at this time, given the potential reductions in chronic disease, and our ongoing engagement and support, it is hoped that the coalition can reintroduce the policy proposal, and progress with policy action.

### Strengths and limitations

A key strength of this study was that we were able to interact with nearly all relevant policy officials as part of the research process. To mitigate potential bias introduced through the involvement of government officials as research partners, we interviewed all stakeholders deemed likely to have a substantial influence on relevant policy processes. As with any single-country study, a potential limitation to the transferability of findings is the influence of local contextual factors on policy processes. We based our analyses on well-established political sciences frameworks to assist in identifying influences on policy processes and to maximize transferability of policy lessons to other contexts (Walt *et al.*, 2008). We strengthened our policy analysis by interlacing of constructs and ideas from a number of policy frameworks (Howlett *et al.*, 2016).

Policy analysis as a method can be highly subjective. Triangulation and the engagement of multiple research disciplines (including public health, political science and health economics) were critical to ensure robust analysis (Walt *et al.*, 2008). Reflexivity was especially important to study design, given the team were engaged to involve and influence the policy process. Given this, when interacting with all stakeholders as part of the research process, we exercised transparency about the nature of our engagement and our intended influence on outcomes. We believe that our team composition (including academic researchers and policymakers) capitalized on the value of a combined insider–outsider position, in that the insiders (policymakers) offered insight to the context, while the outsiders (the academic researchers) introduced new perspectives (Walt *et al.*, 2008). We explicitly identified local contextual factors, and focused on opportunities for lesson drawing as applicable to other contexts.

## Conclusions

This study supported an advocacy coalition in the Solomon Islands to take up a window of opportunity to advocate for an SSB tax. We worked with policymakers to develop a nuanced view of the policy problem, which included that SSB taxes could deliver substantial economic and health benefits to the Solomon Islands, and provided timely advice towards meeting policy objectives that were sensitive to local contextual factors and constraints. The study provided valuable insight into the practical challenges faced by policymakers from lower-resource settings in implementing one of many global food policy recommendations, including that health policymakers find it difficult to demonstrate the potential efficacy of interventions and to operationalize technical aspects of policy design. It also contributed to the development of research methods for action-oriented, trans-disciplinary policy analysis research conducted in real-world policy contexts, to build the knowledge that can accelerate food policy implementation.

Applied prospectively, policy analysis can be utilized to formulate an effective response to policy problems before policy actions are carried out. Findings from this study also highlight that the implementation of global policy recommendations is an ongoing effort for countries, and that research engagement delivered over the longer term is likely to be most effective for supporting implementation. We suggest that there is substantial scope for researchers to more effectively engage with policy advocates to inform and shape real-world health policy improvements.

## Supplementary data

Supplementary data are available at *Health Policy and Planning* online.

## Supplementary Material

czab031_Supp
