# Supplementary material for: Action-oriented prospective policy analysis to inform the adoption of a fiscal policy to reduce diet-related disease in the Solomon Islands
Source: Health Policy Plan. 2021 Apr 7;36(8):1257–68. doi: 10.1093/heapol/czab031 (PMC8428604; doi:10.1093/heapol/czab031)
Supplement: czab031_Supp [file czab031_supp.zip › Figure 1.docx]

Figure 1: Logic model for health effects of SSB tax
