# Supplementary material for: Action-oriented prospective policy analysis to inform the adoption of a fiscal policy to reduce diet-related disease in the Solomon Islands
Source: Health Policy Plan. 2021 Apr 7;36(8):1257–68. doi: 10.1093/heapol/czab031 (PMC8428604; doi:10.1093/heapol/czab031)
Supplement: czab031_Supp [file czab031_supp.zip › Supplementary file.docx]

# Supplementary material: SSB Tax Economic Modelling

## Methods

### Intervention description

Based on discussions with health policymakers and international literature, it has been recommended that a tax rate of at least 20% be implemented to achieve population health benefits (Nakhimovsky et al., 2016) (Nakhimovsky et al., 2016; World Health Organisation, 2016). We therefore modelled a 20% and 40% tax. A 50% pass-through of a 20% tax (equal to 10%) was also modelled to account for manufacturers or retailers not passing on the full amount of the tax. The comparator to the intervention is assumed to be ‘do-nothing’.

### Intervention effectiveness

Due to lack price elasticity (PE) estimates from the Solomon Islands, we sought a PE value that was most relevant. A review of the international literature on price elasticity (PE) estimates from low- and middle-countries found that PE values tend to be higher in low and middle-income countries compared to high-income countries, as consumers are more price sensitive (Green et al., 2013). The most recent systematic review of taxes on SSBs in middle-income countries reported a range of SSB price elasticities between -0.6 to -1.2, (Nakhimovsky et al., 2016) which is slightly lower than a global meta-analysis of PEs for SSBs (-1.3 [95% CI: -0.09 – 0.3])(Cabrera Escobar, Veerman, Tollman, Bertram, & Hofman, 2013). For the Solomon Islands we selected a PE of -0.9 based on the mean of the highest and lowest Pes, which aligned to guidance from the Secretariat of Pacific Communities (SPC)(Teng, 2015). Milk and 100% juice are seldom consumed in the Solomon Islands, thus this analysis did not consider substitution to other (non-taxed) beverages.

### Study population

We modelled the lifetime impact on the Solomon Islands population aged 2-100 years in 2013. Population numbers were obtained from the Household Income and Expenditure survey 2012/2013 (SPC Statistical Division, 2013).

### Health benefit modelling

#### Overview

We used a recently developed model (developed to determine the cost effectiveness of obesity interventions in Australia) to estimate how an SSB tax in the Solomon Islands could change the prevalence of overweight and obesity and the number of cases and deaths from obesity-related diseases. The obesity related health impacts were modelled using Solomon Islands data where available. Changes in body mass index (BMI) were modelled based on projected changes in SSB consumption. Based on the changed in BMI, differences in health adjusted life years (HALYs) and in 9 diseases caused by obesity were predicted (Figure 1). Several methodological adaptations were made for Solomon Islands use (outlined below).

Figure 1: Logic model for health effects of SSB tax

#### Consumption of SSBs

Baseline intake of SSBs for adults was based on data from the Ministry of Health’s NCD STEPS survey 2015 (Minstry of Health & WHO, 2017) by sex and age. Average intake of SSBs by adults was 2 servings per week (340ml serve). For children, data from the Global School Based Health Survey (GSHS) 2011 (World Health Organisation, 2011) indicating that 44% of children had consumed an SSB (assuming serving size of 250ml), one or more times a day. To estimate intake for children aged 2-9 years the daily SSB consumption was assumed to be 3.5 times less than school aged children, as was the case in the Australian Health Survey (Australian Bureau of Statistics, 2015). These were converted to daily consumption in millilitres for each age category (Table 1).

Table 1: Consumption of SSBs

|  | **Men** | | | **Women** | | |
| --- | --- | --- | --- | --- | --- | --- |
| (years) | Overall mean weekly serves**^a^** | Ready to drink SSBs mean daily consumption, mls | Powder mix mean daily consumption, g diluted | Overall mean weekly serves**#** | Ready to drink SSBs mean daily consumption, mls | Powder mix mean daily consumption, g diluted |
| 18-29 | 2.6 | 85 | 47 | 2.8 | 79 | 51 |
| 30-44 | 2.1 | 61 | 38 | 2 | 64 | 36 |
| 45-69 | 1.3 | 33 | 24 | 1.1 | 40 | 20 |
| Children^b^ | Mean daily intake SSBs^b^ |  | | | | |
| 2-9 | 34g |  |  |  |  |  |
| 10-17 | 119g |  |  |  |  |  |
| ^a^ Estimates based on Ministry of Health’s NCD STEPS survey 2015 (Minstry of Health & WHO, 2017)  ^b^ Estimates based on Global School Based Health Survey (GSBHS) 2011 (World Health Organisation, 2011) | | | | | | |

Annual weighted household acquisition (grams) of relevant drinks were obtained from the 2012/13 Household Income and Expenditure Survey (SPC Statistical Division, 2013). This includes items that were purchased, gifted or home-sourced (Table 2). The weighted household acquisition of SSBs was used to estimate the amount of powdered drink mixes consumed as a proportion of all SSBs (37%). This was necessary due to the different caloric content of these drinks. The reductions in quantities of SSBs consumed were converted to average kilojoule equivalents using nutrient tables NUTTAB (Food Standards Australia and New Zealand, 2019).

Table 2: Annual weighted Household acquisition of beverages (grams) and kilojoule equivalents

| **Category** | **Brands** | **Annual weighted acquisition (grams)^b^** | **Kilojoule/ 100ml** |
| --- | --- | --- | --- |
| Fruit juice drink | Juice (fresho,orange,mango etc.) | 79,215,287 | 184^c^ |
| Cordial | Cordial (golden circle, orange: 750ml plastic bottle) | 28,216,245 | 148^c^ |
| Carbonated sugar sweetened | Pacific split | 4,501,084 | 174^c^ |
|  | Soft drink (solbrew mango/pineapple: 285ml bottle) | 129,476,184 |  |
|  | Sprite | 28,623,569 |  |
|  | Szeba | 212,223,407 |  |
|  | Fanta | 55,563,371 |  |
|  | Coca cola | 89,904,950 |  |
|  | Daisy (450g plastic/can) | 9,244,107 |  |
| Chocolate powder drink | Ovaltine | 139,123 | 233^a^ |
|  | Milo | 39,579,607 | 233^a^ |
| 3-in-1 | Tea mix (3 in 1) | 137,292,814 | 178^a^ |
|  | Coffee mix (3 in 1) | 206,664,200 | 198^a^ |
| Powder mixes | Powder drinks (raro, popdrink) | 5,195,513 | 148^c^ |
| ^a^ As prepared, using averages from instructions on sachets purchased in Honiara  ^b^ 2012/13 Household Income and Expenditure Survey (SPC Statistical Division, 2013)  ^c^ Nutrient tables NUTTAB (Food Standards Australia and New Zealand, 2019), | | | |

#### Effect of the tax on body weight

Estimated changes in body weight for adults were calculated based on published relationships between changes in energy expenditure and body weight at the population level. For example, changes in body weight for adults were based on a change of 100 kilojoules per day equalling a 1-kg change in weight, taking 3 years to achieve the total weight change (Kevin D. Hall et al., 2011).

Specific formulas were used for children aged 6-17 years based on the literature (K. D. Hall, Butte, Swinburn, & Chow, 2013) (Table 3). The changes in weight were converted to changes in BMI using average Solomon Island height and weight by gender from the STEPS survey 2015 (Minstry of Health & WHO, 2017) and the (GSBHS) 2011 (World Health Organisation, 2011).

Table 3: Daily reduction in energy expenditure and body weight required for 1kg reduction in body weight

|  | **Kj per kg** |
| --- | --- |
| Adults | 100.0 |
| Boy average age 5 | 232.2 |
| Boy average age 12 | 138.1 |
| Girl average age 5 | 213.4 |
| Girl average age 12 | 130.5 |
| Source: Hall et al (Kevin D. Hall et al., 2011) | |

We modelled the lifetime impact on the current population aged 2-100 years. Potential impact fractions were used to quantify the proportional reduction in disease incidence that would occur if a population were subject to a counterfactual exposure to a risk factor because of an intervention. Disease-specific mortality and morbidity were then combined with all other causes of mortality and morbidity from the population to estimate the total morbidity and mortality in the total population. Disease epidemiology was based on a study of the US burden of diseases, injuries, and risk factors in 2010 (GBD 2010 Country Collaboration, 2013) with substitutions made for Solomon Island prevalence, incidence for diabetes, colon, breast and kidney cancer, hypertensive heart disease, ischaemic heart disease, osteoarthritis and all-cause mortality from the Global Health Data Exchange in 2016 (Institute for Health Metrics and Evaluation, 2016).

We adjusted Australian case fatality rates of the diseases by a factor of 2.6 based on the difference in the probability of dying between ages of 30 and 70 in the Solomon Islands compared to Australia from WHO NCDs country profiles (World Health Organisation, 2014). Population numbers were obtained from the Household Income and Expenditure survey 2012/2013 (SPC Statistical Division, 2013).

The CRE obesity model calculates incremental HALYs and incremental disease incidence. We modelled the SSB tax as a population-based intervention, that is, lifetime health effects of an SSB tax from the altered population distribution of BMI, from all ages (2-100 years). For individuals aged 2<19 years, who do not experience the included diseases, the disability related to obesity itself was quantified, using the health-related quality of life lost due to obesity before and after the intervention, based on the difference between quality-adjusted life year (QALY) weights (Chen et al., 2014).

### Identification of intervention costs

Intervention costs were based on a model of predicted US costs of a SSB tax, using Solomon Island costs and population data (Long et al., 2015). Costs were assessed from both a government and industry perspective for a typical year. Salary ranges were sourced from a 2016 Remuneration Report on the Solomon Islands (Strategic Pay Ltd, 2016) and a Public Service Commission report (Solomon Islands Public Service Commission, 2014).

Included in the costs were administration and compliance costs (for example tax department administration and auditing). Administration costs to the beverage industry were assumed to be equal to the costs to government, based on sales tax evidence in the US (Long et al., 2015). Not included in these costs are the passing of legislation in parliament as this was not within the scope of this report. All costs are reported in 2017 Solomon Island dollars (SBD) unless otherwise indicated (Table 4).

### Modelling of potential healthcare cost savings

Comprehensive estimation of the healthcare costs savings was not possible, however as an indication we calculated the potential savings from hospital admissions of diabetes based on a comprehensive study of health service utilisation in the Solomon Islands by Monash University in 2014 (Centre for Health Economics Monash University & World Bank, 2015). This study was able to estimate an average cost per admission based on costs of all health facilities in the Solomon Islands**.**

### Modelling of tax revenue

We modelled a 20% and 40% tax and 50% pass-through of a 20% tax (equal to 10%)(to account for manufacturers or retailers not passing on the full amount of the tax). We modelled the predicted post-tax consumption, assuming that consumers responded to the tax and population consumption was reduced (using the PE of -0.9).

Predicted tax paid due to the introduction of a 20% SSB tax was calculated as the post-tax mean quantity demanded of each category of SSB consumed, multiplied by 20% of the current retail price. The estimated consumption was calculated based on volumes of household acquisition data from the Household Income and Expenditure survey (SPC Statistical Division, 2013).

In addition to estimating the potential revenue generated from a tax on all SSBs, we modelled the additional potential revenue generated if the tax were extended to also include all frozen ices due to the apparent high consumption them. Using STEPs and GYHS data (Table 1) we generated population annual averages for self-reported consumption of SSBs in litres (ready to drink) or grams (powder mixes). Prices per litre and gram were multiplied by the tax per litre or gram. See Appendix for surveyed prices.

### Uncertainty and sensitivity analysis

The impact of uncertainty around input values on the main outcome measures were estimated by Monte-Carlo simulations (Table 4). Means and 95% uncertainty intervals for the BMI effects on HALYs and intervention costs were reported based on 2,000 iterations using Ersatz version 1.3 software.

Table 4: Input parameters and uncertainty ranges

| **Parameters** | **Value and uncertainty range** | **Source** |
| --- | --- | --- |
| **Health benefit** | | |
| Daily intake of SSB | refer Table 1  Normal distribution of gender and age specific means | STEPS survey 2015 and Global School Based Health Survey. |
| Own price elasticity of demand | -0.9 (Standard errors calculated based on Z score with alpha 0.1 two tailed (probability 0.05) | Literature review of similar countries (Cabrera Escobar et al., 2013; Nakhimovsky et al., 2016) |
| Change in kj to change in weight | Refer Table 3 | Hall et al(Kevin D. Hall et al., 2011) |
| **Cost of implementing a SSB tax** | | |
| Administration and compliance time costs per million people (FTE, government and industry) | 0.32 FTE (0.10-0.54) | Long et al [46]. Administration and compliance time costs per million people (FTE, government and industry) |
| Field audit time costs per million people per year for (FTE, government and industry) | 0.30 FTE (0.24- 0.35) | Long et al [46]. Field audit time costs per million people per year for (FTE, government and industry) |
| Field audit direct costs per million people per year for (government and industry) | $10,300 (13,800-17,200) | Long et al [46]. Samples drawn from a gamma distribution. (5th Percentile $10,300 95th percentile $17,200) based on an estimate of field audit direct cost |
| Accountant yearly salary (government and industry) | $50,790  (33,954-66,500) | Strategic Pay Ltd (Strategic Pay Ltd, 2016) , level 11/8, Public Service Commission report (Solomon Islands Public Service Commission, 2014) |
| **Calculation of Tax Revenue** | | |
| 3-in-1 powder (coffee, tea) annual acquisition undiluted (population) | 343,957,014 grams | Household Income Expenditure Survey *(SPC Statistical Division, 2013)* |
| Carbonated soft drinks annual acquisition (population) | 529,536,671 grams | Household Income Expenditure Survey *(SPC Statistical Division, 2013)* |
| Frozen ices annual acquisition (population) | 335,488,801 grams | Household Income Expenditure Survey *(SPC Statistical Division, 2013)* |
| Juice drinks, cordial, flavoured powders annual acquisition (population) | 112,627,045 grams | Household Income Expenditure Survey *(SPC Statistical Division, 2013)* |
| Liquid Beverages, average daily intake (population) | 67 grams | Estimate based on Ministry of Health’s NCD STEPS survey 2015 (Minstry of Health & WHO, 2017)  and Global School Based Health Survey (GSBHS) 2011 (World Health Organisation, 2011) |
| Powdered Beverages, average daily intake | 36 grams | Estimate based on Ministry of Health’s NCD STEPS survey 2015 (Minstry of Health & WHO, 2017)  and Global School Based Health Survey (GSBHS) 2011 (World Health Organisation, 2011) |
| 3-in-1 powder (coffee, tea) price | SBD$1.50, 20g | Bus Stop Store, Honiara, Solomon Islands on 21/2/18 |
| Carbonated soft drinks price | SBD$20.00-$24.24 per litre (ref Appendix) | Bus Stop Store, Honiara, Solomon Islands on 21/2/18 refer Appendix |
| Frozen ices 100ml price | SBD$1.50, 100ml | Bus Stop Store, Honiara, Solomon Islands on 21/2/18 |
| Juice drinks, cordial | SBD $20.00- 24.00 per litre | Bus Stop Store, Honiara, Solomon Islands on 21/2/18 refer Appendix |
| Flavoured powders | SBD $3.00, 25g | Bus Stop Store, Honiara, Solomon Islands on 21/2/18 |
| Population 2 years and over in 2013 | 582,177 | (SPC Statistical Division, 2013) |

## Limitations of economic modelling

As with all economic models, the model results represent the best estimate of a potential effect in the absence of stronger direct evidence. The model does not incorporate the effects of changes in SSB consumption on oral health or indirect costs, such as reduced productivity due to absenteeism and disability, which means that the societal savings from the tax are likely to be substantially underestimated.

The modelling in a Solomon Islands context required a number of assumptions due to lack of data but are based on best-available evidence. Due to data availability, the healthcare cost savings were only calculated for diabetes, resulting in an underestimate of the potential healthcare cost savings. Information on SSB consumption rates was ascertained through analysis of 3 different data sources (HIES, STEPs, GSHS). Each of the surveys used as a measure of SSB consumption (STEPS, GSHS and HIES) had a different methodology and measure for consumption, and they are not usually comparable. This led to a large difference in reported intake. We used conservative estimates for the health impact modelling and high and low estimates of tax revenue. There are inherent limitations of self-reported survey data, and underreporting remains a key limitation (Gemming, Jiang, Swinburn, Utter, & Mhurchu, 2014). Use of household acquisition data as a proxy for food, energy and nutrient intake cannot account for intra-household distribution (Molteldo, Nathalie Troubat, Michael Lokshin, & Sajaia, 2014; Sahal Estime, Lutz, & Strobel, 2014).

The extent of substitution to other beverages (cross-price elasticities) were not available, and thus were also not included, although juice and milk are rarely consumed in the Solomon Islands and a switch to diet beverages would have a similar impact as replacement with water.

### Appendix 1: Beverage prices in the Solomon Islands

| **Ready to drink SSBs** | **Brand** | **Size** | | **Price** | | **Price per litre** |
| --- | --- | --- | --- | --- | --- | --- |
| Szeba cola/orange/pine | Szetu | 300ml | | $5.00 | | $16.67 |
| Fruit Juice - mango/orange | Marigold | 250ml | | $6.00 | | $24.00 |
| Fruit Juice - mango/orange | Drinho | 250ml | | $6.00 | | $24.00 |
| cheers - orange /strawberry | Cheers | 325ml | | $6.00 | | $18.18 |
| Fanta – all flavours | Fanta | 330ml | | $8.00 | | $24.24 |
| Coca cola - can | Coca cola | 375ml | | $8.00 | | $21.33 |
| Coca cola - bottle | Coca cola | 500ml | | $10.00 | | $20.00 |
| BU Energy | Coca cola | 440ML | | $10.00 | | $22.73 |
| Ole /freso | Ole | 200ml | | $3.00 | | $15.00 |
| C2 green tea/lemon tea/peach | C2 | 500ml | | $12.00 | | $24.00 |
| Average Price per litre | | | | | | $21.00 |
| 40% Tax amount per litre based on average price | | | | | | $8.00 |
| 20% Tax amount per litre based on average price | | | | | | $4.00 |
| **Powder mix SSBs** | | | | | | **Price per gram** |
| Coffee Mix 3-in-1 sachet | Super | 20g | | $1.50 | | $0.08 |
| Coffee Mix 3-in-1 sachet | Good day | 20g | | $1.50 | | $0.08 |
| Milk tea 3-in-1 sachet | Super | 20g | | $1.50 | | $0.08 |
| Master Tea 3-in-1 sachet | Master | 20g | | $1.50 | | $0.08 |
| Milo 3-in-1 sachet | Nestle | 33g | | $4.00 | | $0.20 |
| Hot chocolate sachet | Aik Cheong | 40g | | $4.00 | | $0.20 |
| Cappuccino sachet | Aik Cheong | 40g | | $4.00 | | $0.20 |
| White coffee sachet -blue | Aik Cheong | 30g | | $4.00 | | $0.20 |
| Tang sachet | Tang | 25g | | $3.00 | | $0.15 |
| Average Price per gram | | | | | | $0.14 |
| 40% Tax amount per gram based on average price | | | | | | $0.06 |
| 20% Tax amount per gram based on average price | | | | | | $0.03 |
| **Flavoured Frozen Ice Blocks** | | | | | | **Price per gram** |
| Kool pop | Tuckers | | 74ml | | $6.00 | 0.08 |
| True Love | Tuckers | | 86ml | | $6.00 | 0.07 |
| Ice stick | Homemade | | 100ml | | $1.5 | 0.015 |
|  | Average price per gram | | | | | $0.06 |
| 40% Tax amount per g based on average price | | | | | | 0.02 |
| 20% Tax amount per g based on average price | | | | | | 0.01 |

*Source: Bus Stop Store, Honiara, Solomon Islands on 21/2/18*

ReferencesUncategorized References

Australian Bureau of Statistics. (2015). Australian Health Survey 2011-12. Canberra, Australia.

Cabrera Escobar, M. A., Veerman, J. L., Tollman, S. M., Bertram, M. Y., & Hofman, K. J. (2013). Evidence that a tax on sugar sweetened beverages reduces the obesity rate: a meta-analysis. *BMC Public Health., 13:1072.*(doi), 10.1186/1471-2458-1113-1072.

Centre for Health Economics Monash University, & World Bank. (2015). Solomon Islands Health Facilities Costing Study.

Chen, G., Ratcliffe, J., Olds, T., Magarey, A., Jones, M., & Leslie, E. (2014). BMI, health behaviors, and quality of life in children and adolescents: a school-based study. *Pediatrics, 133*(4), e868-874. doi: 10.1542/peds.2013-0622

Food Standards Australia and New Zealand. (2019). Australian Food Composition Database. Retrieved 11 February, 2020, from <http://www.foodstandards.gov.au/science/monitoringnutrients/nutrientables/nuttab/Pages/default.aspx>

GBD 2010 Country Collaboration. (2013). GBD 2010 country results: a global public good. *Lancet, 381*(9871), 965-970. doi: 10.1016/s0140-6736(13)60283-4

Gemming, L., Jiang, Y., Swinburn, B., Utter, J., & Mhurchu, C. N. (2014). Under-reporting remains a key limitation of self-reported dietary intake: an analysis of the 2008/09 New Zealand Adult Nutrition Survey. *Eur J Clin Nutr, 68*(2), 259-264. doi: 10.1038/ejcn.2013.242

Green, R., Cornelsen, L., Dangour, A. D., Turner, R., Shankar, B., Mazzocchi, M., & Smith, R. D. (2013). The effect of rising food prices on food consumption: systematic review with meta-regression. *BMJ., 346*, f3703.

Hall, K. D., Butte, N. F., Swinburn, B. A., & Chow, C. C. (2013). Dynamics of childhood growth and obesity: development and validation of a quantitative mathematical model. *Lancet Diabetes Endocrinol, 1*(2), 97-105.

Hall, K. D., Sacks, G., Chandramohan, D., Chow, C. C., Wang, Y. C., Gortmaker, S. L., & Swinburn, B. A. (2011). Quantification of the effect of energy imbalance on bodyweight. *The Lancet, 378*(9793), 826-837. doi: 10.1016/S0140-6736(11)60812-X

Institute for Health Metrics and Evaluation. (2016). Global Health Data Exchange. Retrieved 9 Feb, 2018, from <http://ghdx.healthdata.org/gbd-results-tool>

Long, M. W., Gortmaker, S. L., Ward, Z. J., Resch, S. C., Moodie, M. L., Sacks, G., . . . Claire Wang, Y. (2015). Cost Effectiveness of a Sugar-Sweetened Beverage Excise Tax in the U.S. *Am J Prev Med, 49*(1), 112-123. doi: 10.1016/j.amepre.2015.03.004

Minstry of Health, & WHO. (2017). Solomon Islands NCD Risk Factors STEPs report. Solomon Islands.

Molteldo, A., Nathalie Troubat, Michael Lokshin, & Sajaia, Z. (2014). Analyzing Food Security Using Household Survey Data. Washington DC: World Bank,.

Nakhimovsky, S. S., Feigl, A. B., Avila, C., O'Sullivan, G., Macgregor-Skinner, E., & Spranca, M. (2016). Taxes on Sugar-Sweetened Beverages to Reduce Overweight and Obesity in Middle-Income Countries: A Systematic Review. *PLoS One., 11*(9), e0163358. doi: 0163310.0161371/journal.pone.0163358. eCollection 0162016.

Sahal Estime, M., Lutz, B., & Strobel, F. (2014). Trade as a structural driver of dietary risk factors for noncommunicable diseases in the Pacific: an analysis of household income and expenditure survey data. *Global Health, 10*, 48. doi: 10.1186/1744-8603-10-48

Solomon Islands Public Service Commission. (2014). Solomon Islands Public Service Unified Salary Structure 1st July 2014-December 2014.

SPC Statistical Division. (2013). Solomon Islands 2012/13 Household Income and Expenditure Survey.

Strategic Pay Ltd. (2016). Solomon Islands Renumeration Report.

Teng, A. (2015). *Sugar-sweetened beverage tax in Pacific Island countries and territories: A discussion paper*.

World Health Organisation. (2011). Global school-based student health survey (GSHS). Retrieved 9 Feb, 2018, from <http://www.who.int/ncds/surveillance/gshs/solomondataset/en/>

World Health Organisation. (2014). Solomon Islands. Noncommunicable diseases country profiles 2014

World Health Organisation. (2016). Fiscal policies for diet and prevention of noncommunicable diseases: technical meeting report Geneva, Switzerland.
