# Supplementary material for: Action-oriented prospective policy analysis to inform the adoption of a fiscal policy to reduce diet-related disease in the Solomon Islands
Source: Health Policy Plan. 2021 Apr 7;36(8):1257–68. doi: 10.1093/heapol/czab031 (PMC8428604; doi:10.1093/heapol/czab031)
Supplement: czab031_Supp [file czab031_supp.zip › Table 1.docx]

Table 1: Mean SSB consumption in the Solomon Islands (2012), modelled change in SSB consumption, change in weight and impact on health outcomes

|  | | | | | **Lifetime change in incident cases for existing population^a^** | | | |
| --- | --- | --- | --- | --- | --- | --- | --- | --- |
| Modelled tax rate | Mean intake SSBs per person (g/day) | Change in SSB consumption (g/day) | Change in weight (kg) | **Total HALYs gained** (95% UI) | **Cancers^b^** (95% UI) | **Heart disease and stroke^c^**   (95% UI) | **Type 2 Diabetes** (95% UI) | **Osteoarthritis**  (95% UI) |
| **10%** (50% pass-through) | 88 | -4.1 | -0.06 | 312 (265 to 367) | -35 (-99 to 12) | -322 (-364 to -280) | -6,517  (-7,632 to -5,392) | -3,833 (-4,888 to -2,707) |
| **20% tax** | 88 | -8.0 | -0.13 | 606 (510 to 724) | -69 (-194 to 20) | -623 (-701 to -550) | -12,692 (-14,822 to -10,519) | -2,009 (-2,567 to -1438) |
| **40% tax** | 88 | -15.2 | -0.24 | 1,149 (973 to 1,360) | -132 (-363 to 35) | -1,180 (-1,320 to -1,056) | -24,138 (-28,080 to -20,083) | -1,027 (-1,327 to -722) |
| Negative numbers indicate a reduction; UI: Uncertainty Interval; HALYs: Health adjusted life years;^a^ based on 2013 population size;^b^ Cancers include colon, breast, endometrial and kidney  ^c^ Heart disease includes hypertensive heart disease, ischemic heart disease;  SSBs: Sugar sweetened beverages  HALYs: Health adjusted life years | | | | | | | | |
