# Supplementary material for: Action-oriented prospective policy analysis to inform the adoption of a fiscal policy to reduce diet-related disease in the Solomon Islands
Source: Health Policy Plan. 2021 Apr 7;36(8):1257–68. doi: 10.1093/heapol/czab031 (PMC8428604; doi:10.1093/heapol/czab031)
Supplement: czab031_Supp [file czab031_supp.zip › Table 2.docx]

Table 2: Estimated revenue generation from a SSB tax in the Solomon Islands at different tax rates

|  | Annual Household acquisition (grams) | Total consumption after 20% tax^a^ | Tax revenue at 20% tax  (SBD) | Total consumption after 40% tax^a^ | Tax revenue at 40% tax  (SBD) |
| --- | --- | --- | --- | --- | --- |
|  |  |  | $4/L (liquid SSB)^b^  $0.03/g (powder SSB)^b^  $0.01/g (ices)^b^ |  | $8/L (liquid)  $0.06/g (powder)  $0.02/g (ices) |
| 3-in-1 powder (coffee, tea) | 343,957,014 | 282,044,751 | 7,897,253 | 220,132,488 | 12,327,419 |
| Carbonated soft drinks | 529,536,671 | 434,220,070 | 1,823,724 | 338,903,469 | 3,647,448 |
| Frozen ices | 335,488,801 | 275,100,816 | 3,301,209 | 214,712,832 | 6,602,419 |
| Juice drinks, cordial, flavoured powders | 112,627,045 | 92,354,176 | 387,887 | 72,081,308 | 775,775 |
| Chocolate powder drinks | 40,386,522 | 33,116,948 | 927,274 | 25,847,374 | 1,854,549 |
| Total (SBD) | **1,361,996,053** | **1,116,836,763** | **14,337,349** | **871,677,473** | **25,207,611** |
| Total (USD) | **163,439,526** | **134,020,412** | **1,720,482** | **104,601,297** | **3,024,913** |
| ^a^ Based on price elasticity of demand for SSBs of -0.9  ^b^ Modelling rate established based on surveyed price for that type of SSB  SBD= Solomon Island Dollar  1 SBD= 0.12 USD (as at February 2018) | | | | | |
