# Supplementary material for: Action-oriented prospective policy analysis to inform the adoption of a fiscal policy to reduce diet-related disease in the Solomon Islands
Source: Health Policy Plan. 2021 Apr 7;36(8):1257–68. doi: 10.1093/heapol/czab031 (PMC8428604; doi:10.1093/heapol/czab031)
Supplement: czab031_Supp [file czab031_supp.zip › Table 3.docx]

Table 3: Design implications for SSB tax implementation in the Solomon Islands context

| Policy options | | Implications for implementation |
| --- | --- | --- |
| 1 | Target all products coded under a selection of HS codes | Relatively simple to administer because of the adoption of the Automated System of Customs Data and thus the ability to ‘flag’ products for special treatment (e.g. special taxes).  Requirements:   - Review of the HS codes to ensure that all ‘problematic’ SSBs are captured by targeted codes (e.g. beverages section and dairy section) - ‘Splicing’ (disaggregation) of HS codes to avoid flagging non-SSBs within existing HS codes (e.g. sweetened coffee mix is targeted, but instant coffee is not), generating codes for all beverages with added sugar. |
| 2 | Target all beverages under a selection of HS codes containing more than a certain percentage of sugar, as defined by a nutrient threshold | Uses nutrient composition information to identify products containing sugar content above a designated threshold  Requirements:   - Splicing of HS codes to define codes with sugar content above nominal threshold), with compliance checks by trained nutrition professionals - Mandatory display of nutrient composition data to the exterior of shipping crates and cartons^a^ - Training to enable customs officials to interpret nutrition composition panels and nutrient thresholds. |
| 3 | Target of all beverages with any ‘added sugar’ or ‘free-sugar’ in the ingredients list | Uses ingredients lists to classify food.  Requirements:   - Mandatory display of ingredients lists on manifest shipping crates and carton exterior^a^ - Review of the HS codes to ensure that all ‘problematic’ SSBs were captured under a set of codes being targeted - Training to enable customs officers to identify sugar- containing beverage products (i.e. sugar added under different ingredient names) |
| 4 | Apply a volumetric tax based on sugar content by volume | A variable tax based on sugar content and product volume  Requirements:   - Mandatory display of nutrient composition data to the exterior of shipping crates and cartons^a^ - Training to enable customs officers to undertake detailed calculations to determine the rate of tax |
| 5 | Apply an ad valorem tax based on product value | A tax applied to the product value outlined on the manifest  Requirements:   - Splicing of HS codes to avoid flagging non-SSBs within existing HS codes (e.g. sweetened coffee mix is targeted, but instant coffee is not) |
| ^a^ These could possibly be addressed by requiring the exporting country to outline nutrition composition or ingredients lists of each product in the shipping manifest.  HS Codes: Codes applied under the international recognised Harmonised Commodity Description and Coding system  SSB: Sugar Sweetened Beverages include all liquid and powdered beverages (including carbonated, milk-based, flavoured powders, cordials and juice drinks) that have been sweetened with any form of added sugar. | | |
