# Supplementary material for: Action-oriented prospective policy analysis to inform the adoption of a fiscal policy to reduce diet-related disease in the Solomon Islands
Source: Health Policy Plan. 2021 Apr 7;36(8):1257–68. doi: 10.1093/heapol/czab031 (PMC8428604; doi:10.1093/heapol/czab031)
Supplement: czab031_Supp [file czab031_supp.zip › Table 4.docx]

Table 4: Summary of recommendations on SSB tax design

| Objective of the tax | To reduce consumption of SSBs in the Solomon Islands, particularly in the population groups with the highest consumption (e.g. children and adolescents, urban dwellers), and prevent growth in consumption among the rural population. |
| --- | --- |
| Definition and identification of beverages to be targeted | New HS codes are spliced (disaggregated) by a group of health and customs experts, so that all beverages with ‘added sugars’ are included. Added sugars can be identified through the ingredients list and declaration on manifest. |
| Tax collection point | Excise is equally applied to imported and locally produced drinks by import excise and manufacturers excise, aligning to other ‘health taxes’- tobacco and alcohol excise |
| Rate of tax | 20%-40%  Consider applying 20% to liquid beverages and 40% on powder beverages |
| Tax mechanism | A volumetric tax, applied as a tax per Litre (for liquids) or per gram (for powders).  20% tax is equal to: SBD$4/L or SBD 0.03/g  40% tax is equal to: SBD $8/L or SBD 0.06/g |
| Monitoring | Establish a SSB tax monitoring and evaluation plan to collect baseline and ongoing beverage pricing and consumption information, to convey trends in pricing, purchasing and tax revenue |
| Additional policy changes | Removal of import exemptions on SSBs to MSG countries, and the addition of SSBs to the exemptions list for PICTA.  Implementation of mandatory ingredients labelling, to be visible to customs officers on import |
| HS Codes: Harmonised Commodity Description and Coding system  MSG: Melanesian Spearhead Group  PICTA: Pacific Island Countries Trade Agreement  SBD: Solomon Islands Dollar  SSB: Sugar Sweetened Beverages include all liquid and powdered beverages (carbonated, milk-based, flavoured powders, cordials and juice drinks) that have been sweetened with any form of added sugar. | |
